# Impact of Adjuvant Chemotherapy on FIGO Stage I Ovarian Clear Cell Carcinoma: A Systematic Review and Meta-Analysis

**DOI:** 10.3389/fonc.2022.811638

**Published:** 2022-05-17

**Authors:** Min Yin, Jiaxin Yang, Huimei Zhou, Qian Liu, Sijian Li, Xinyue Zhang

**Affiliations:** Department of Obstetrics and Gynecology, National Clinical Research Center for Obstetric and Gynecologic Diseases, Peking Union Medical College Hospital, Chinese Academy of Medical Sciences and Peking Union Medical College, Beijing, China

**Keywords:** adjuvant chemotherapy, clear cell ovarian carcinoma, stage I, survival, systematic review and meta-analysis

## Abstract

**Background:**

Ovarian clear cell carcinoma (OCCC) is an uncommon subtype of epithelial ovarian carcinoma (EOC) that is often diagnosed at an earlier stage in younger women. It remains uncertain whether adjuvant chemotherapy improves the prognosis of patients with stage I OCCC.

**Objective:**

This systematic review and meta-analysis aimed to assess the impact of adjuvant chemotherapy on survival in patients with stage I OCCC.

**Search Strategy:**

Eligible studies were screened from PubMed, Web of Science, Embase, and the Cochrane Library up to October 10, 2021.

**Selection Criteria:**

Studies that compared the oncological outcomes of adjuvant chemotherapy with observation were included.

**Data Collection and Analysis:**

Six studies comprising a total of 4553 patients were enrolled in our study, of whom 3320 (72.9%) patients had undergone adjuvant chemotherapy and 1233 (27.1%) had not.

**Main Results:**

The 5-year disease-free survival (DFS) and 5-year overall survival (OS) of stage I OCCC were 82.7% and 86.3%, respectively. In the overall population, adjuvant chemotherapy did not improve the 5-year DFS (83.2% vs 83.7%, OR 0.77, 95% CI 0.21-2.82, P=0.69) or 5-year OS (87.3% vs 83.6%, OR 1.30, 95% CI 0.86–1.98, P=0.22). Further subgroup analysis on stage IA/IB suggested that adjuvant chemotherapy did not impact 5-year DFS (OR 0.20, 95% CI 0.01-5.29, P=0.34) or 5-year OS (OR 1.52, 95% CI 0.78-2.98, P=0.22). For stage IC including 1798 patients, adjuvant chemotherapy revealed a significant survival benefit for 5-year OS (84.5% vs 83.3%, OR 1.44, 95% CI 1.08-1.94, P=0.01). Furthermore, the administration of adjuvant chemotherapy was found to be associated with a better 5-year OS (OR 4.98, 95% CI 1.12-22.22, P=0.04) in stage IC2/3. But no inferences regarding the effect of AC on stage IC2/3 can be made due to the limited size of the non-AC arm.

**Conclusion:**

This study indicated that adjuvant chemotherapy did not improve the prognosis of stage IA and IB OCCC patients. However, for patients with stage IC, due to the retrospective, heterogenous and older data with limited sample size, the pooled results of our study should be interpreted with caution. More prospective studies on the role of adjuvant chemotherapy in stage I OCCC are warranted.

**Systematic Review Registration:**

PROSPERO, CRD42021287749.

## Introduction

Ovarian cancer is the third most common cancer among women worldwide, and is the leading cause of gynecological cancer death (1). Ovarian cancer is associated with high mortality rates because most women are diagnosed at an advanced stage. Only approximately 30% of patients with ovarian cancer are diagnosed with early-stage disease, which is localized to the gynecologic organs and has not spread to adjacent structures in the pelvis or the upper abdomen (2). The International Federation of Gynecology and Obstetrics (FIGO) stage I disease is confined to one or both ovaries (3). FIGO stage I is subdivided into three stages. In stage IA or IB, the disease is confined to one or both ovaries with intact capsules and no malignant cells in the ascites or peritoneal washings, respectively. Stage IC indicates tumor limited to one or both ovaries and can be divided into three subgroups: IC1, surgical spill; IC2, capsule ruptured before surgery or tumor on ovarian surface; IC3, malignant cells in the ascites or peritoneal washings. The treatment of early-stage disease, which means FIGO stage I and IIA, involves surgical removal of all visible disease, often followed by adjuvant chemotherapy. Adjuvant chemotherapy is used in order to eradicate any microscopic deposits of tumors that may remain after surgery and to reduce the risk of recurrence (4). Nevertheless, 10%-50% of patients who receive surgery for the treatment of early-stage ovarian cancer have recurrence, and these recurrences are often resistant to various forms of salvage treatment (5).

Over 85% of ovarian cancers are epithelial ovarian cancer (EOC). EOC can be classified into five main histologic subtypes based on microscopic features: serous, endometrioid, clear cell, and mucinous. Ovarian clear cell carcinoma (OCCC) is a less common subtype accounting for approximately 5-13% of EOC in Western populations, but its incidence is much higher in Asia reaching 20-25% of all EOC (6). Studies and clinical experience have shown that OCCC is a unique pathological type with unique pathogenesis, molecular changes, and clinical behavior. Endometriosis is believed to be a risk factor for OCCC, and is estimated to be present in more than 50% of cases (7). Women diagnosed with OCCC are usually younger and at an earlier stage than patients with high-grade serous ovarian cancer (HGSOC). The overall survival rate of patients with early-stage OCCC is favorable. However, the prognosis of advanced-stage or relapsed disease is much worse due to chemoresistance (8).

The question of whether platinum-based adjuvant chemotherapy can improve the prognosis of patients with early-stage epithelial ovarian cancer is important, and several clinical trials was performed. International Collaborative Ovarian Neoplasm 1 (ICON1) and Adjuvant ChemoTherapy In Ovarian Neoplasm (ACTION) compared platinum-based adjuvant chemotherapy with observation following surgery in early-stage ovarian cancer. A total of 925 patients, were randomly assigned to receive platinum-based adjuvant chemotherapy or observation, 130 of whom had OCCC. This study indicated that platinum-based adjuvant chemotherapy improved overall survival and recurrence-free survival at 5 years, but in subgroup analyses within the subcategories of histologic cell type, no difference was found (9). According to the European Society for Medical Oncology (ESMO) and European Society of Gynecological Oncology (ESGO) guidelines (10), no adjuvant chemotherapy could be considered in OCCC patients with FIGO stage IA-IC1 disease with complete surgical staging. However, according to the latest US National Comprehensive Cancer Network (NCCN) guidelines, intravenous platinum-based therapy is recommended to all stage I OCCC patients, while observation could be considered only in patients with stage IA OCCC.

The role of adjuvant chemotherapy in FIGO stage I OCCC is still under debate. Several studies have compared oncologic outcomes between women with stage I OCCC who received adjuvant chemotherapy and those who did not, however, there are inconsistencies in previous study results (11, 12). Moreover, the small number of patients included in these retrospective studies limited the generalizability of the findings. In this article, we aimed to systematically review the literature on adjuvant chemotherapy in stage I OCCC and to perform a meta-analysis in order to determine whether adjuvant chemotherapy would improve overall survival and prolong recurrence-free survival in women with stage I OCCC.

## Method

This systematic review and meta-analysis were conducted following the Preferred Reporting Items for Systematic Reviews and Meta-Analyses (PRISMA) guidelines. A protocol was submitted before the search, including populations, interventions, comparators, outcomes, and the study designs, registered at the International Prospective Register of Systematic Reviews (PROSPERO, CRD42021287749). Populations were women with surgically stage I OCCC; intervention was chemotherapy following surgery; comparator was observation following surgery; and outcomes were 5-year disease-free survival (DFS) and 5-year overall survival (OS).

### Search Strategy

Two investigators independently searched all the related studies in databases, including PubMed, Web of Science, Embase, and the Cochrane Library until October 10, 2021. The search terms included “clear cell ovarian cancer”, “clear cell ovarian carcinoma”, “early stage”, “stage I”, “adjuvant chemotherapy”, “chemotherapy following surgery” and “postoperative chemotherapy”, which were combined using the Boolean operators “AND” or “OR”.

### Selection Criteria

Studies meeting the following criteria were included (1): original studies focusing on survival outcomes of adjuvant chemotherapy and observation in stage I OCCC (2); the study designs included retrospective cohort studies, case-control studies, or randomized controlled trials (3); 5-year DFS and 5-year OS could be extracted directly or indirectly from the study; and (4) full text available in English.

The exclusion criteria were as follows (1): duplicate data (2); paper without reporting original data (3); unavailable full text in English; and (4) in vivo and/or in vitro studies. Two independent investigators looked through the titles and abstracts of the articles and then carefully read the full text to determine the final included articles.

The resulting studies were examined and selected at three levels. Leve 1 selected via titles and article abstracts. The two authors studied all the articles independently. Relevant articles were further checked at the full-text level. Level 3 was a critical examination of the selected studies.

### Quality Assessment

The quality of the eligible studies was assessed by the Newcastle Ottawa Scale (NOS). A study can be given a maximum of one star for each numbered item within the selection and outcome categories and a maximum of two stars within the comparability category. A quality score was determined for each study, with six or more stars considered to be of high quality. Inconsistency between the two investigators was settled by discussion.

### Data Extraction

Data were extracted independently by two reviewers, and discrepancies were settled by discussion. The following information was extracted from each eligible study: first author, year of publication, study country, study design, study period, number of patients in each arm, completeness of surgical staging, regimen and duration of chemotherapy given in the treatment arm, time of follow-up, 5-year DFS, 5-year OS and study quality.

### Statistical Analysis

RevMan 5.3 (Cochrane Review software) was used to perform statistical analysis including pooling the data and producing the forest plots. We used odds ratios (ORs) as the summary statistic to statistically analyze the dichotomous outcomes, reported with 95% confidence intervals (CIs), and P<0.05 was considered statistically significant. The I^2^ value and Q test were used to assess heterogeneity. If I^2^ values were less than 50% or P values were larger than 0.1, the data were calculated using fixed-effects models. Otherwise, we used the random-effects model. Subgroup analysis based on substages was used to deeply explore the heterogeneity and its potential effect. Publication bias was assessed by a funnel plot created by RevMan 5.3 software.

## Results

### Identification and Selection

**Figure 1:**
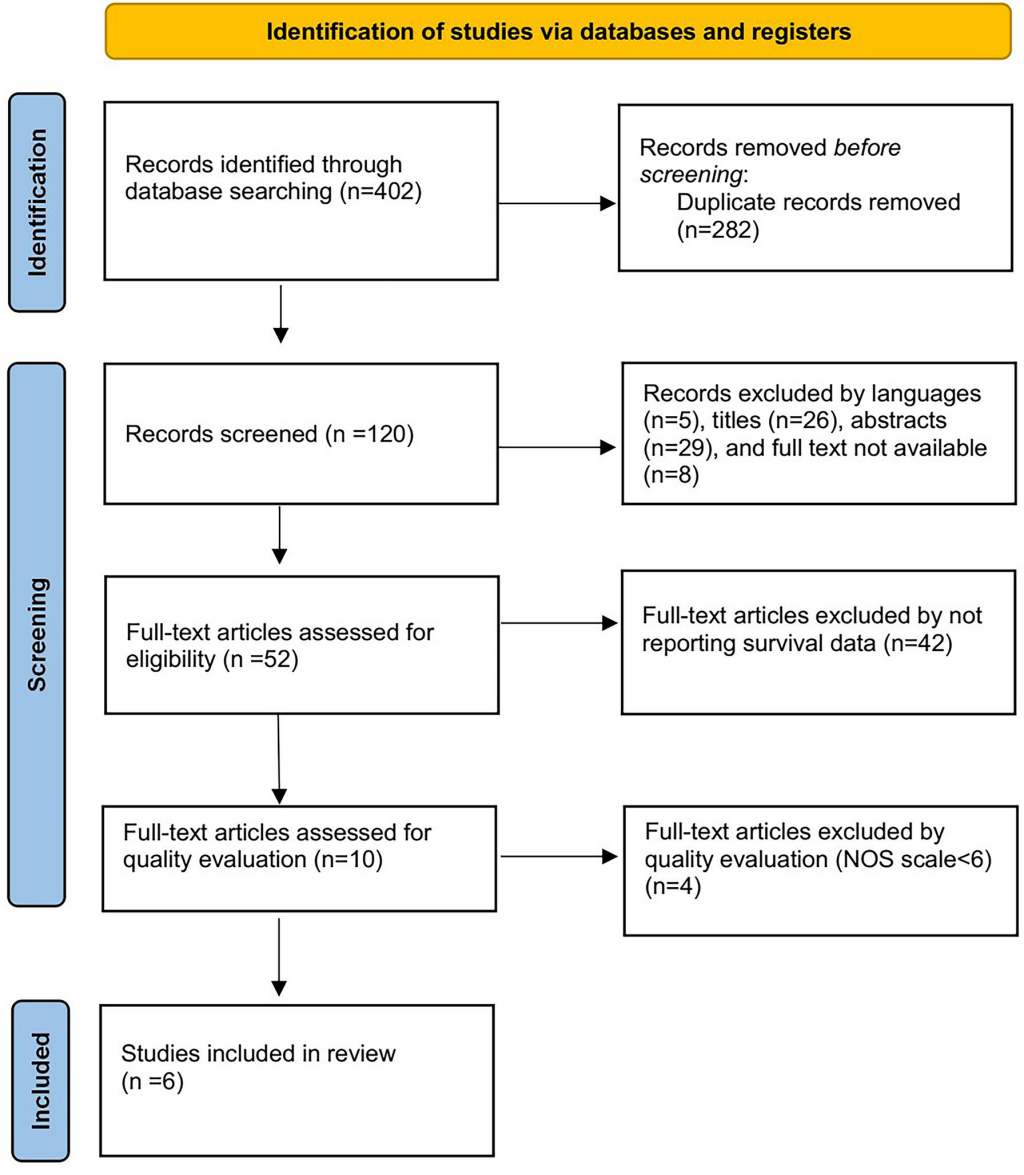
Flow chart of selection process.

Four hundred and two studies were initially obtained from the online database. After title and abstract screening of these articles, we identified 52 articles. Full-text screening excluded 42 articles for not reporting survival data and 4 articles with a low NOS scale smaller than six. Finally, the remaining 6 studies were included for further analysis (11–16). The specific selection process is shown in **Figure 1**.

### Characteristics of Retrieved Studies

Of the 6 studies, all were retrospective cohort studies. The study period ranged from 1991-2015. Three studies were conducted in Japan, while two studies were conducted in the USA and 1 was conducted in Canada. A total of 4553 patients were enrolled for the meta-analysis: 3320 (72.9%) underwent adjuvant chemotherapy, and 1233 (27.1%) underwent observation. Five studies reported data on the completeness of surgical staging: 2484 (54.6%) patients underwent complete surgical staging procedures and 1995 patients were not reported. Four studies reported the regimen of chemotherapy, which was as follows: paclitaxel and platinum; paclitaxel and carboplatin; cyclophosphamide, doxorubicin and cisplatin; cyclophosphamide and cisplatin; irinotecan and cisplatin; and single agent carboplatin, for three to six cycles after an initial surgery. The median follow-up time ranged from 30 months to 3.18 years. The main characteristics and outcomes of each study are shown in **Table 1**.

**Table 1:** Main characteristics and outcomes of the included studies.

Study	Country	Study type	Study period	Total patient number	Patients with complete surgical staging (%)	Group	Number of patients (%)	Median age (y)	Regimen of chemotherapy	Cycles of chemotherapy	Follow-up time	5-year DFS	5-year OS	NOS scale
Takano et al. 2010 (13)	Japan	RC	1992-2005	219	145(66.2%)	AC	195(89.0%)	52	Paclitaxel and platinum; cyclophosphamide + doxorubicin + cisplatin; cyclophosphamide + cisplatin; irinotecan and cisplatin	3-6	48(7-160) mo	77.0%	86.0%	7
						non-AC	24(11.0%)	57	–	–	43(8-98) mo	96.0%	100.0%	
Mizuno et al. 2012 (14)	Japan	RC	1991-2007	134	134(100%)	AC	91(67.9%)	53	Taxane + carboplatin; platinum-based therapies	3-6	64(14-190) mo	87.9%	91.1%	8
						non-AC	43(32.1%)	51	–	–	60(7-191) mo	97.5%	100.0%	
Takada et al. 2012 (15)	Japan	RC	2000-2009	73	73(100%)	AC	30(41.1%)	53.5	Paclitaxel + carboplatin; irinotecan-based therapies	NR	30(14-113) mo	80.1%	87.4%	8
						non-AC	43(58.9%)	54	–	–	56 (13–119) mo	73.9%	81.7%	
Hogen et al. 2017 (16)	Canada	RC	1995-2014	60	60(100%)	AC	29(48.3%)	54.5	Paclitaxel + carboplatin; paclitaxel + cisplatin; single agent carboplatin	2-8	6.21 yrs	86.2%	92.8%	8
						non-AC	31(51.7%)	54.7	–	–	3.18 yrs	58.0%	73.6%	
Oseledchyk et al. 2017 (11)	USA	RC	2000-2013	1995	NR	AC	1346(67.5%)	–	NR	NR	64 mo	–	85.0%	7
						non-AC	649(32.5%)	–	–	–	64 mo	–	83.0%	
Nasioudis et al. 2018 (12)	USA	RC	2004-2015	2072	2072(100%)	AC	1629(78.6%)	–	NR	NR	59.1(1.1-151.4) mo	–	89.2%	7
						non-AC	443(21.4%)	–	–	–	68.3(1.7-151.8) mo	–	82.6%	

AC, adjuvant chemotherapy; DFS, disease-free survival; mo, months, yrs, years; NOS, Newcastle-Ottawa quality assessment; OS, overall survival; RC, retrospective cohort study.

### Synthesized Findings

**Figure 2:**
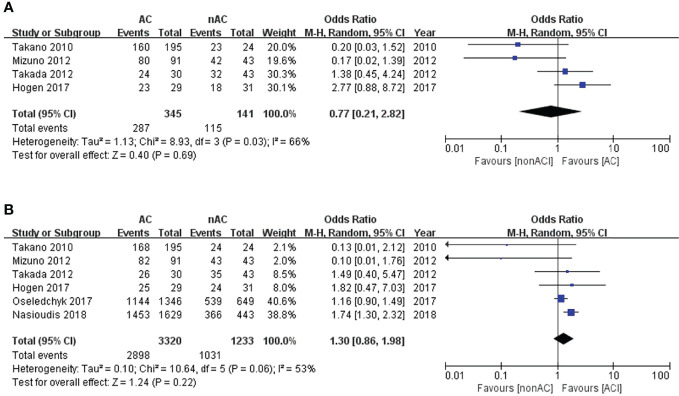
Forest plot of the meta-analysis in patients with stage I OCCC, **(A)** 5-year DFS, **(B)** 5-year OS.

The 5-year DFS and 5-year OS of stage I OCCC were 82.7% and 86.3%, respectively. Four studies (13–16) reported 5-year DFS, involving 345 patients in the adjuvant chemotherapy (AC) group and 141 patients in the non-AC group. We found that adjuvant chemotherapy did not improve the 5-year DFS, with 83.2% compared to 83.7% of the non-AC group (OR 0.77, 95% CI 0.21-2.82, P = 0.69; I^2 ^= 66%; **Figure 2A**). Additionally, 5-year OS data were retrievable from all six studies (11–16). No significant difference in 5-year OS was observed between the two groups (87.3% vs 83.6%, OR 1.30, 95% CI 0.86-1.98, P=0.22; I^2 ^= 53%; **Figure 2B**).

**Figure 3:**
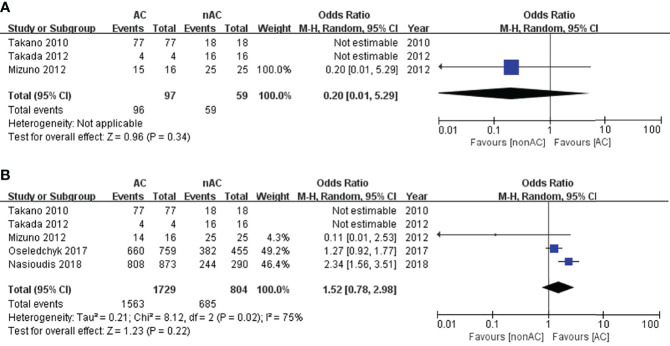
Forest plot of the meta-analysis in patients with stage IA/IB OCCC, **(A)** 5-year DFS, **(B)** 5-year OS.

Since we observed significant heterogeneity among the studies, we performed subgroup analysis based on substages to deeply explore the heterogeneity and its potential effect. In the subgroup stage IA/IB, adjuvant chemotherapy did not impact 5-year DFS (OR 0.20, 95% CI 0.01-5.29, P=0.34; **Figure 3A**) or 5-year OS (OR 1.52, 95% CI 0.78-2.98, P=0.22; **Figure 3B**).

**Figure 4:**
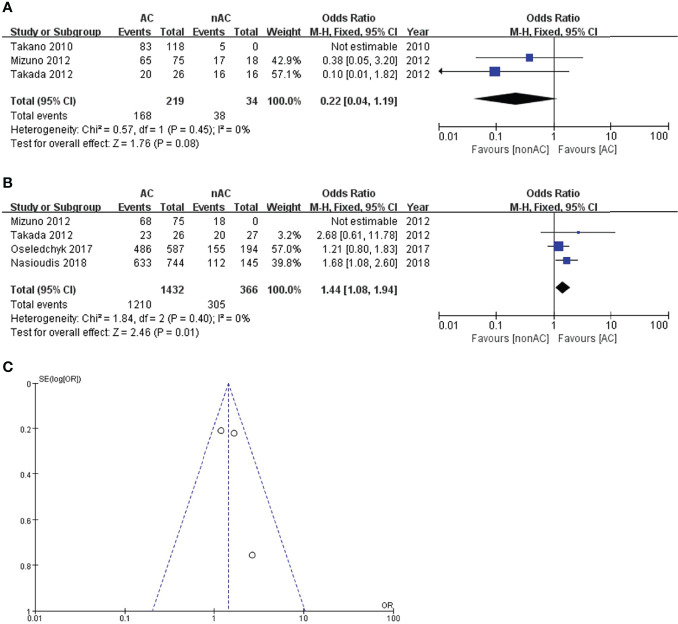
Forest plot of the meta-analysis in patients with stage IC OCCC, **(A)** 5-year DFS, **(B)** 5-year OS. **(C)** Funnel plot of 5-year OS in stage IC.

For subgroup stage IC, adjuvant chemotherapy did not improve 5-year DFS (OR 0.22, 95% CI 0.04-1.19, P=0.08; **Figure 4A**). Four studies (11, 12, 14, 15) reported the 5-year OS data of stage IC. This sub-analysis included 1798 patients: 1432 (79.6%) received adjuvant chemotherapy and 366 (20.4%) did not receive adjuvant chemotherapy. Compared with the non-AC group, the pooled OR was 1.44 with a 95% CI of 1.08 to 1.94 in the AC group (**Figure 4B**), which revealed a significant survival benefit for 5-year OS. Patients with adjuvant chemotherapy experienced a 5-year OS of 84.5% compared with 83.3% in the non-AC group (P=0.01). Moreover, there was no heterogeneity (I^2 ^= 0). The funnel plot of 5-year OS in stage IC was symmetrical, and all the studies analyzed were within the 95% CI, suggesting no serious publication bias among the eligible studies (**Figure 4C**).

**Figure 5:**
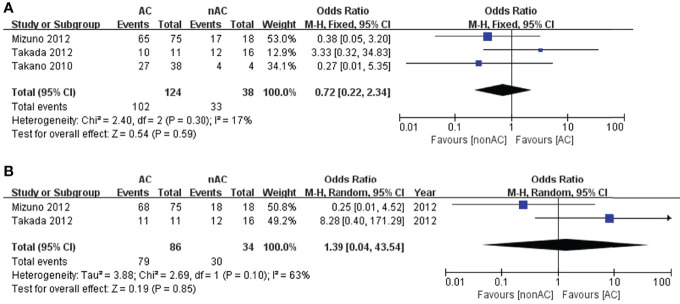
Forest plot of the meta-analysis in patients with stage IC1 OCCC, **(A)** 5-year DFS, **(B)** 5-year OS.

Since we observed that adjuvant chemotherapy significantly improved the 5-year OS of patients with stage IC OCCC, we conducted sub-analysis among stage IC. Three studies reported survival data in stage IC1. The pooled results reported no improvement in comparison to observations in terms of 5-year DFS (OR 0.72, 95% CI 0.22-2.34, P=0.59; **Figure 5A**) and 5-year OS (OR 1.39, 95% CI 0.04-43.54, P=0.85; **Figure 5B**).

**Figure 6:**
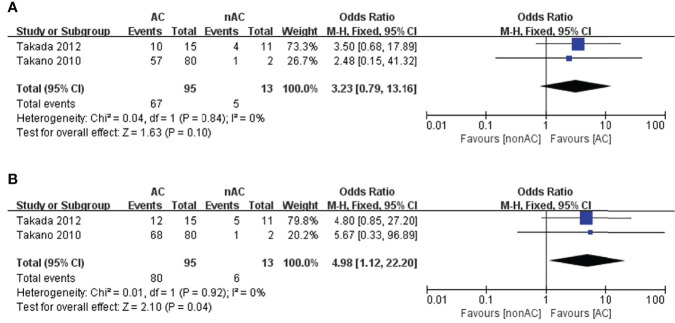
Forest plot of the meta-analysis in patients with stage IC2/3 OCCC, **(A)** 5-year DFS, **(B)** 5-year OS.

As for stage IC2/3, the number of patients that underwent observation was significantly lower than those who were treated with AC. This probably represents more aggressive use of AC in those with surface involvement, and positive cytology. The administration of adjuvant chemotherapy was found to be associated with a better 5-year OS (OR 4.98, 95% CI 1.12-22.22, P=0.04; **Figure 6B**), but a slightly improvement in term of 5-year DFS (OR 3.23, 95% CI 0.79-13.16, P=0.10; **Figure 6A**). However, only two studies (13, 15) reported the survival data in stage IC2/3, with only 13 patients in the non-AC arm. No inferences regarding the effect of AC on stage IC2/3 can be made due to the limited size of the non-AC arm.

## Discussion

In our meta-analysis, six studies on the role of adjuvant chemotherapy in stage I OCCC were eligible. Our meta-analysis with pooled outcomes of 4553 patients revealed that adjuvant chemotherapy did not improve the 5-year DFS and 5-year OS of stage IA and IB OCCC patients. A better 5-year OS was observed in the AC arm of stage IC2/3, but the numbers of events in the non-AC arm were so small that the significance is unknown. What’s more, the benefits for AC in stage IC were based on retrospective, heterogenous and older data with limited sample size. For this reason, the results of our study should be interpreted with caution.

The initial therapy for EOC includes surgery and adjuvant therapy. Surgical cytoreduction to R0 is the mainstay of treatment, followed by adjuvant chemotherapy. Completeness of surgical staging is an independent prognostic factor (17). The randomized clinical trial called ACTION was initiated to test the efficacy of adjuvant chemotherapy in patients with early-stage ovarian cancer. This study found that patients in the observation arm who were optimally staged had statistically significantly better overall and recurrence-free survival than patients who were non-optimally staged, suggesting that adjuvant chemotherapy in early-stage ovarian cancer may work predominantly by affecting small-volume or microscopic tumor implants or metastases that remain unnoticed at the time of surgical staging. Lawrie et al. (18) performed a systematic review including randomized controlled trials of adjuvant chemotherapy versus observation after surgery in women diagnosed with early-stage EOC. They found that adjuvant chemotherapy improved survival and reduced the risk of ovarian cancer recurrence compared with observation after initial surgery. However, it remained uncertain whether women with stage I OCCC would benefit much from adjuvant chemotherapy as data were not consistently reported by this subgroup in the included studies.

Since OCCC only represents approximately 10% of all ovarian carcinomas, there is limited evidence addressing the role of adjuvant treatment for early-stage patients specifically for OCCC. Compared to other subtypes of EOC, patients with OCCC are relatively insensitive to conventional platinum-based chemotherapy, showing only an 11% to 27% response rate, while the response rate of patients with high grade serous ovarian cancer is 73% to 81% (19). Currently, the NCCN recommends adjuvant platinum-based chemotherapy to all stage I OCCC patients, while observation could be considered only in patients with stage IA OCCC. In contrast, according to the ESMO/ESGO guidelines, it could be omitted in stage IA-IC1 patients after comprehensive surgical staging. This again underlines the importance of thorough and comprehensive surgical staging. The role of adjuvant chemotherapy is quite interlinked with the completeness of surgical staging in women with apparent early-stage EOC. The quality of surgical staging is associated with the reliability of the diagnosis of early-stage disease. Systematic pelvic and para-aortic lymphadenectomy is necessary for accurate staging because the survival of women with stage III disease is worse than that of women with true stage I or II disease.

Specifically, for OCCC, there is different evidence on the role of adjuvant treatment for stage I patients. In our analysis, four included studies confined their investigation among patients who had received comprehensive surgical staging. Mizuno et al. (14) retrospectively evaluated 185 patients with stage I OCCC and they indicated that adjuvant chemotherapy did not contribute to the improving prognosis of stage IA and IC1 OCCC. Takada et al. (15) reported similar survival outcomes in their retrospective analysis of 73 patients with stage I OCCC who underwent complete surgical staging. In stage IA OCCC, four of the patients underwent chemotherapy, whereas the remaining 16 patients received no additional therapy. No recurrence was observed in either group. Of the patients with stage IC, no statistical difference in PFS and OS between the two groups. However, a large cohort study, including 1629 patients in the AC group and 443 patients in the non-AC group, analyzed the data of stage I OCCC patients from the National Cancer Database. The AC group had a better 5-year OS than the non-AC group (89.2% vs 82.6%, P<0.001), suggesting that adjuvant chemotherapy could be associated with a survival benefit for patients with stage I OCCC (12). Since 2012, the British Columbia Cancer provincial treatment guidelines no longer recommend adjuvant treatment for stage IA-IC1 OCCC patients. Liu et al. (20) retrospectively analyzed the oncological outcomes of all stage I OCCC patients since policy implementation and observed that adjuvant therapy could be safely omitted in OCCC patients with stage I A/B and IC1 disease.

Regarding adjuvant chemotherapy regimens, ESMO and ESGO guidelines recommend that patients with early-stage disease receive carboplatin alone or carboplatin and paclitaxel. While NCCN guidelines show that the preferred regimen is carboplatin and paclitaxel, other recommended regimens are carboplatin and liposomal doxorubicin or docetaxel and carboplatin. The GOG 157 trial compared three versus six cycles of adjuvant paclitaxel and carboplatin chemotherapy in patients with high-risk, early-stage ovarian cancer, indicating that three additional cycles of chemotherapy added toxicity without significantly reducing the risk of cancer recurrence. Moreover, *post-hoc* analysis of this trial suggested that the subset of patients with high-grade serous histology benefit from six cycles of chemotherapy, but that OCCC histology does not (21). Currently, the need for adjuvant chemotherapy in stage I EOC, including OCCC, after comprehensive staging surgery is being evaluated by the Japanese Gynecologic Oncology group in a randomized phase III trial (JGOG3020). The results of JGOG3020 may provide solider evidence on the role of adjuvant chemotherapy in patients with stage I OCCC.

The benefit of adjuvant radiation therapy (RT) in patients with early-stage OCCC has been evaluated in a series of studies. Especially for OCCC, the beneficial effect of adjuvant RT may be more pronounced because of its unique mode of spread, most cases confined to the pelvis, and relatively resistant to traditional chemotherapy (CT). A retrospective study by Swenerton et al. (22) explored the influence of ovarian cancer histotype on the effectiveness of RT and reported an enhanced curability of patients with stage I and II clear cell, endometrioid, and mucinous histotypes by RT-containing adjuvant therapy. Another retrospective study (23) on 241 patients with stage I-II OCCC indicated that the delivery of abdominopelvic irradiation after 3 cycles of carboplatin and paclitaxel had no discernible survival benefit for patients with stage IA and IC1, whereas for a subset of high-risk patients defined as stage IC2/3 and stage II, it improved 5-year DFS by 20%. Nevertheless, Hogen et al. (24) reported that adjuvant RT was not associated with a survival benefit in patients with stage I and II OCCC. Therefore, considering these disputable results, both the ESMO/ESGO and NCCN guidelines do not considered adjuvant RT as part of the therapeutic strategies for early stage OCCC.

The strength of our study is that we pooled six studies, collecting a large number of patients up to 4553, and performed further subgroup analysis to summarize the current evidence on the question of whether postoperative chemotherapy is beneficial to stage I OCCC patients. Nonetheless, the present study has certain limitations that should be noted. First, there was heterogeneity among studies because there were various factors in the baseline characteristics, such as fully staged surgery, number of chemotherapy cycles, chemotherapy agents and the exact dosage. Second, inherent biases could not be avoided because all the included studies in our meta-analysis were retrospective cohort studies instead of randomized controlled trials, which may affect the quality of the evidence. Finally, it was not possible to evaluate the impact of various chemotherapy regimens and cycles on patient survival because these data are not comparable in the included papers.

## Conclusion

The results of our meta-analysis suggested that adjuvant chemotherapy had little effect on the survival of stage IA and IB OCCC patients. However, for patients with stage IC, due to the retrospective, heterogenous and older data with limited sample size, the results of our meta-analysis should be interpreted cautiously. Owing to the inherent biases of the studies included in the meta-analysis, more prospective studies on the role of adjuvant chemotherapy in stage I OCCC are warranted. Furthermore, prospective trials randomizing patients with stage IC2/3 OCCC to AC and nonAC group with or without pelvic RT should be considered to assess postoperative adjuvant treatment for early stage OCCC.

## Data Availability Statement

The original contributions presented in the study are included in the article/supplementary material. Further inquiries can be directed to the corresponding author.

## Author Contributions

JY, HZ, and MY, designed the study and reviewed the final manuscript. QL and SL, literature search and data collection. XZ and MY, statistical analysis and writing the manuscript. All authors contributed to the article and approved the submitted version.

## Funding

This work was supported by the Chinese Academy of Medical Sciences Initiative for Innovative Medicine (CAMS-I2M-1-002).

## Conflict of Interest

The authors declare that the research was conducted in the absence of any commercial or financial relationships that could be construed as a potential conflict of interest.

## Publisher’s Note

All claims expressed in this article are solely those of the authors and do not necessarily represent those of their affiliated organizations, or those of the publisher, the editors and the reviewers. Any product that may be evaluated in this article, or claim that may be made by its manufacturer, is not guaranteed or endorsed by the publisher.
